# The Experience of Caring for a Medically Complex Child in the Neonatal Intensive Care Unit: A Qualitative Study of Parental Impact

**DOI:** 10.3390/children12020123

**Published:** 2025-01-23

**Authors:** Natascia Bertoncelli, Martina Buttera, Elisa Nieddu, Alberto Berardi, Licia Lugli

**Affiliations:** 1 Neonatal Intensive Care Unit, Women’s and Children’s Health Department, University Hospital of Modena, 41100 Modena, Italy; 2 Department of Medical and Surgical Sciences for Mother, Children and Adults, Postgraduate School of Pediatrics, University of Modena and Reggio Emilia, 41124 Modena, Italy

**Keywords:** children with medical complexity, parents’ experiences, relationships with healthcare professionals

## Abstract

Background: Parents of children with life-limiting or life-threatening illnesses and/or medical complexity experience intense stress and pain soon after the birth and lifelong. Understanding parents’ experiences and coping strategies is the prerequisite to provide tailored support to families. **Aim:** To explore the experiences of parents of children with medical complexity (CMCs) during hospitalization in a Family-Centered Care (FCC) neonatal unit and after discharge. **Design:** Qualitative study. Methods: Semi-structured interviews were administered to the parents of children with medical complexity admitted to the Neonatal Intensive Care Unit (NICU) of Modena between October 2016 and January 2024. The interview was developed based on three time points: birth, hospitalization, and discharge, focusing on parents’ experiences, emotions, and communication with healthcare professionals. The interviews were analyzed using the template analysis. **Results:** A total of 10 parents were interviewed. Four domains were identified, encompassing eight significant themes in the parents’ experiences and their communication with healthcare professionals. The relevant emotions included anxiety and fear for survival, fatigue, and guilt over the child’s suffering, alongside hope and trust that parents felt entitled to nurture. Relationships with professionals were characterized by expectations and frustrations; mothers and fathers had different perceptions and reactions to the situation they were facing. **Conclusions:** This qualitative study explores the experiences of parents of CMCs in a neonatal intensive care unit adopting FCC. From admission to discharge, parents’ emotions were influenced by the child’s unique clinical history. Active listening and the humane attitude of healthcare professionals were the aspects most appreciated by parents.

## 1. Introduction

Children with medical complexity (CMCs) often experience conditions that impair neurodevelopment, leading to chronic behavioral and cognitive challenges. These children typically have severe, long-term health issues, rely heavily on healthcare systems, and require significant resources. Their care demands specialized skills and a multidisciplinary team approach, both during hospitalization and after discharge. Advances in medical technology have improved survival rates, resulting in an increasing number of CMCs, many of whom need intensive care from birth, often in the Neonatal Intensive Care Unit (NICU), where life-saving interventions are provided.

Parents of CMCs play a crucial role in managing their child’s care, a task that can be emotionally overwhelming. They are responsible for complex medical procedures, managing their child’s symptoms and pain, and striving to ensure the best possible quality of life. Healthcare professionals must understand the emotional burden parents carry and offer tailored support. The perception and emotional experience of parents when a medically complex condition is detected can vary significantly depending on whether the condition is identified in utero or at birth, as well as whether the child is preterm or full-term. These differences can influence the parents’ psychological and emotional responses, decisions about care, and long-term outlooks for their child.

As it stands, qualitative studies reveal common parental concerns, such as the emotional impact of the condition, the difficulties of disease management, and the social context in which the family operates [1,2,3,4,5,6,7]. For instance, a study of parents with children suffering from spinal muscular atrophy (SMA) identified key concerns, including how the diagnosis is communicated, the sufficiency of information provided, and ongoing anxiety and uncertainty. Recommendations for healthcare providers include limiting the number of professionals involved in communications, providing clear written and visual information, and encouraging the presence of a trusted support person during medical visits [8]. Parents of CMCs often feel uncertain about their child’s diagnosis and treatment, expressing dissatisfaction with how the diagnosis is delivered [9]. The lack of active listening by healthcare professionals can erode trust and complicate relationships [10]. Effective communication and collaboration are therefore essential for building trust. Family-Centered Care (FCC) is an approach that fosters collaboration between families and healthcare teams, emphasizing mutual respect, shared decision-making, and the inclusion of parents as key partners in the care process [11,12,13,14]. Caring for CMCs requires coordinated efforts from various specialists, including neonatologists, pediatricians, nurses, physiotherapists, psychologists, and social workers. These professionals not only manage the child’s medical needs but also provide emotional and psychological support to the family. To improve care for CMCs, healthcare teams must prioritize personalized care plans, symptom management, and the emotional well-being of both the child and the parents.

## 2. Materials and Methods

### 2.1. Aims

The study aimed to understand how parents of CMCs perceived the path from NICU admission to discharge. It also sought to explore their experiences with the communication of their child’s clinical condition and the establishment of a support network at home.

Additionally, the study aimed to define and develop a dedicated multidisciplinary NICU team responsible for the comprehensive care of CMCs throughout their stay, from admission to discharge.

### 2.2. Participants

The parents of 10 full-term CMCs, hospitalized at the NICU of the University Hospital of Modena, Italy, from October 2016 to January 2024, were recruited. During this time period, 35 children with a complex medical condition and a diagnosis of genetic and/or malformative pathology were admitted to the NICU in Modena, excluding cases involving prematurity or hypoxic-ischemic encephalopathy. Twelve children passed away, and the parents of thirteen children declined to participate in the interview due to privacy concerns. The aims of the study were explained through a phone call to parents, and their consent and willingness to participate to the study were collected. This study was approved by the Ethics Committee of the Area Vasta Emilia Nord with the amendment AOU 0001235/24 on 10 April 2024.

The clinical characteristics of the children are shown in Table 1. Information about mothers and fathers who participated in the study are not provided for privacy reasons.

### 2.3. Design

Semi-structured interviews were conducted with the parents of the recruited children. These interviews took place at the NICU of the University Hospital of Modena between April and August 2024, following the children’s discharge. The majority of the interviews were conducted a few years after discharge, with one interview taking place 8 years later and another 3 months after discharge. The NICU at Modena is a reference center for Family-Centered Care (FCC) and also serves as a training center for the NIDCAP method (Newborn Individualized Developmental Care and Assessment Program) [15]. The interviews were conducted by a neonatologist and a physiotherapist who had previously assessed the children as part of the rare disease follow-up program during their first two years of life. Each interview lasted between 30 and 40 min, was audio-recorded, and later transcribed by one of the researchers (MB). The interview guide was developed based on the experience of the two interviewers, as well as a review of relevant literature. Adjustments were made to the guide when it became apparent that certain questions were redundant or not conducive to eliciting meaningful responses from parents. Some questions were combined, and the guide was streamlined accordingly. Despite these changes, the consistency of the content across interviews was maintained (see Table 2). The interviews covered several key themes, including parents’ reactions at birth, during hospitalization, and at discharge, as well as their communication with healthcare professionals.

### 2.4. Data Analysis

Data analysis was performed using the template analysis method. The initial analysis was conducted by one of the researchers (NB), who segmented and coded various meaning units, including significant sentences, paragraphs, and words. This process facilitated the identification of interpretative categories [16,17]. Two researchers (NB and LL) then compared their findings by revisiting the interpretative categories derived from the initial analysis. Through this comparison, eight key themes emerged, reflecting the emotions and experiences of the parents of CMCs in this study. Data saturation was only partially achieved for certain themes, such as “anxiety and fear for survival”. For one theme in particular, “relationship with other parents”, saturation was not reached, leading to the exclusion of this theme from the results.

All interviews were initially translated into English by the first author, then back-translated into Italian and checked for consistency.

## 3. Results

Ten parents participated in the semi-structured interviews: four couples (mother and father, *n* = 4), one father only (*n* = 1), and five mothers only (*n* = 5). One couple and one mother requested translation into their native language through the cultural mediation service offered by the University Hospital of Modena. Four main domains were identified, encompassing eight significant themes: parents’ emotions, relationship with healthcare professionals, relationship within the couple, and outlook on the future (Table 3).

### 3.1. Parents’ Emotions

Emotions are crucial for adapting to life situations. The word “emotion” comes from the Latin *e-moveo* (to move from), indicating a powerful shift in the body and mind. They involve three components: physiological reactions, altered thinking, and behavioral responses. Emotions are classified into fundamental emotions (fear, sadness, joy, anger, surprise, disgust) and learned emotions, shaped by culture. Fundamental emotions are innate, observed from early childhood, and universal; they emerge suddenly, last briefly, and are distinct from one another. They are universally triggered by specific events, like danger causing fear or loss causing sadness. While emotions help us adapt, they can also cause distress when too intense or uncontrollable [18,19].

Since the birth of a CMC is often an unexpected, unpredictable, and threatening event, analyzing emotional experiences is considered an essential aspect of this study.

#### 3.1.1. Anxiety and Fear for Survival

The NICU admission of a child with a potentially life-threatening condition is a profoundly stressful and traumatic experience for parents. The emotional impact of hospitalization, coupled with the uncertainty surrounding the diagnosis and prognosis, overwhelms parents, leading them into a whirlwind of emotions, including immediate fear for their child’s survival. This fear is deep and all-consuming, often accompanied by feelings of helplessness, vulnerability, and uncertainty. Parents feel overwhelmed by an unpredictable future, unable to foresee what lies ahead, and their expectations for the child they had imagined are shattered. As a result, they remain in a constant state of alert, trying each day to brace themselves for the worst. The initial fear for survival can persist for a long time; over time, parents struggle to live with and manage it, striving to find a balance between hope and the harsh reality.

*“I felt fear, worry. I knew she was sick and had some problems; I experienced many emotions at once … It was very hard because right from the start, there was the possibility that my baby wouldn’t survive … Of course, I couldn’t even sleep and kept crying”.*

[Mother 8].

#### 3.1.2. Hope and Trust

At the time of admission and during discussions about their child’s clinical condition, parents emphasized the importance of leaving room for hope. Professionals caring for children with medical complexity (CMCs) often provide concrete information about the potential negative outcomes of the diagnosis, which can undermine a family’s ability to maintain hope for the future. It is not uncommon for healthcare providers to misinterpret a family’s emotional response to hope as a lack of understanding regarding the severity of their child’s condition. However, families can simultaneously accept the devastating reality of the diagnosis while still holding on to hope for the future. There is a delicate balance between delivering clear and direct clinical information about outcomes and preserving hope. Parents placed their trust in the professionals and asserted their right to maintain hope.

*“When she was born, we were relieved to hear a doctor in the NICU say, ‘There are children with trisomy 18 who grow up, and some even live to be 20 years old’. One should never say, ‘Resign yourselves’, as someone told us while we were in that whirlwind. We are not masters of life and death. We must be very careful in delivering news because it doesn’t all depend on the literature. It hurt us to hear doctors say things like, ‘This child has no future’. But each child’s story is unique. Let’s wait and see; we must say, ‘We’ll do our best’. God decides the future. We must be careful not to say, ‘She’s doomed,’ right away. These words weigh heavily, like a boulder”.*

[Father 3]

#### 3.1.3. Guilt and Fatigue

The communication of a rare disease diagnosis creates a profound temporal rift, marking a clear division between “before” and “after”. Parents of children with rare diseases and medical complexities are suddenly confronted with an unpredictable and often daunting future, which evokes intense and frequently painful emotions. The anxiety about what lies ahead, along with the need to reorganize family life after the diagnosis, demands a tremendous amount of emotional and physical energy. Parents often report feeling both exhausted and overwhelmed by the burden of these changes.

*“They took the baby away immediately and said nothing. I could only hear him crying, but they didn’t let us near him. We were confused, scared, and worried. Then, we had a meeting with Dr. […], who explained the treatments in detail, and that helped calm us down. When I saw him for the first time, I felt an overwhelming sense of guilt for what he had, and for what he still faces. I felt guilty for bringing him into the world. Then we waited for the medicine to arrive, and it felt like an eternity”.*

[Mother 5]

### 3.2. Relationship with Healthcare Professionals

Communication is key to building relationships with families, especially when merging complex clinical information with parents’ emotional experiences. For parents of CMCs, clear and empathetic communication is essential, requiring both transparency and sensitivity in tone, words, and timing. Trust, crucial for effective care, is established through these initial interactions. Unspoken expectations, such as how communication is delivered, can lead to frustration if unmet. Active listening is vital; it is about being open, engaged, and understanding the other’s perspective. Active listening fosters trust and meaningful dialogue, contrasting with a detached, neutral approach. It requires emotional engagement and an openness to new insights. Through active listening, we establish relationships of recognition, respect, and mutual learning, which are the conditions necessary to jointly and creatively address the problem at hand. This fits very well when it comes to relationships between healthcare professionals and parents of CMCs.

#### 3.2.1. Expectations

The way in which parents are informed about their child’s daily clinical condition plays a crucial role in shaping the relationship and trust between parents and healthcare professionals. During these discussions, parents value having their perspectives listened to and respected. Acknowledging their emotions, addressing their questions and concerns, and practicing active listening all contribute to a sense of being understood and supported. This approach helps parents feel that professionals are caring for both them and their child.

*“Another positive aspect is that when she was hospitalized here, I was able to come and go while still being able to do things as a mother. You made me feel like a mom, and you always treated me well. […] When she was in the unit, I would call at night, and when they reassured me that everything was fine, I felt at ease. In the morning, I’d wake up not feeling tired; I’d come here eager to be a mom. So, for me, it was definitely a difficult time, but, all in all, it was a good time too—even though it wasn’t what I had expected. It wasn’t just a bad period; there was also the positive aspect of being cared for”.*

[Mother 2]

#### 3.2.2. Frustrations

Parents often experience a deep sense of frustration when they are unable to immediately understand the situation or know how to care for and protect their hospitalized child, especially when faced with an uncertain diagnosis. This sense of confusion is compounded by the complexity of medical terms, unexpected diagnoses, or even simple terms that do not align with their expectations. They may feel lost, unprepared, and without the tools necessary to fully comprehend the situation or act effectively. This can lead to emotional responses, either expressed openly or suppressed. Parents want the best for their children, and any threat to their child’s health or development is perceived as a personal challenge, often resulting in feelings of isolation and despair.

*“I have to say that it requires humility from the professionals. Parents need to receive the right information about each issue. If the right answers can’t be provided, they should refer to other, more specialized centers for the benefit of the patient. […] They told us, ‘It’s all for nothing’. They said, ‘You were heroes for giving birth to her, now be heroes and let her go’. But as long as she’s alive, something can be done. At that time, we were inexperienced. Later, we learned to choose the right people, the doctors to ask for advice. However, overall, we don’t have bad memories”.*

[Father 3]

### 3.3. Relationship Within the Couple

#### Different Perception of Mother and Father

Parents reported experiencing different attitudes and behaviors regarding their child’s journey toward a disease diagnosis. Both parents faced a wide range of emotions when receiving clinical information about their child and throughout the entire hospitalization process. Mothers, in particular, often carried the burden of daily caregiving. At times, fathers were optimistic and hopeful that everything would be resolved, while at other times, they distanced themselves from the child to come to terms with the unexpected diagnosis. These differing responses to the same situation could create tension within the couple. The mother, feeling isolated in her suffering and caregiving duties, would sometimes interpret the father’s detachment as misunderstanding, even though his efforts were often an attempt to cope and maintain control over the situation.

*“Yes, from the beginning to the end, I approached it more calmly. On one hand, I hoped things would go well, but as the doctors kept explaining the situation, I began to have doubts. He (the father) wasn’t as calm; he was somewhat agitated and always hoped that things would work out. […] Even when we had meetings every 2–3 weeks, I always left feeling uncertain about how things would turn out, while he kept saying, ‘You’ll see, she’ll be fine, she’ll grow, she’ll walk, everything will be okay’. But I didn’t believe that. […] I’ve always felt that way, even when they discussed palliative care. I was against it, while her father would have been okay with keeping her like that for life, even if she didn’t get better and perhaps even worsened. On the other hand, I couldn’t bear seeing her in that much pain. Someone always had to be with her, and it didn’t seem fair […] to her little brother”.*

[Mother 2]

### 3.4. Vision of the Future

#### 3.4.1. Daily Life at Home

Parents described the process of adapting to a “new normal” at home as both exhausting and overwhelming. Often, it was the mother who took on the primary responsibility for caring for the child alone, while also having to reorganize daily life and priorities for the entire family. Parents found this adjustment emotionally draining, but sometimes the positive moments with their child—however small—were so rewarding that they helped bear the weight of the disruptions to the routine they had envisioned before their child’s birth.

*“At home, it was a crisis. A crisis because you go from the hospital, where everyone takes care of A, to being at home, where you’re alone because the father has to go to work. A was isolated for a long time, out of fear of infections, so it was very difficult. It was just me and him, all the time. It was very heavy. But once the isolation ended, things got a little easier. We could go out, my parents came to visit, and after that, it improved … Even when we’re at the pool, I watch the other children, see what they’re doing, and I notice the difference—A doesn’t do the same things as the other children. I find it hard to accept that he’s different”.*

[Mother 5]

#### 3.4.2. Social Integration

Parents often envision the moment when their child will have to navigate integration into the broader social environment, whether it’s at school, on the playground, or in any other setting where they interact with peers. They worry about how their child will be received and treated by others. The fear of social integration emerged as one of the most distressing concerns for these parents. This fear is rooted in the anxiety that their child might be excluded or even bullied for being different from others. Parents are especially concerned that teachers, other parents, and children may lack the understanding or sensitivity necessary to support their child’s inclusion and integration.

*“Today, my biggest fear is not so much the medical challenges, but how society will approach him. […] The fear that when he goes to school, he might be bullied. I worry about how society will accept him, how it embraces diversity. I’m afraid of exclusion because of his differences. Now, when I’m out and about, I look at the sidewalks and wonder whether A. will be able to climb them, or how we’ll manage with the stroller... I’m thinking about the medium- and long-term future. I just hope he meets good people who will make sure he lacks nothing”.*

[Mother 5]

## 4. Discussion

Parents of CMCs experience a wide range of emotions during hospitalization. They are deeply committed to receiving clear and accurate information from healthcare professionals about their child’s health, managing their relationship with their partner, and adjusting to daily life after discharge. Four key themes emerged in this study: anxiety and fear, hope and trust, guilt and fatigue, and family organization and social integration. Parents seek transparent, clear communication about their child’s condition, but they also require hope and empathy to avoid despair. Effective communication is crucial, as it can either foster trust and hope or lead to frustration and distrust, depending on how the information is delivered. Our results support those of [20], who found that, during the so-called “information phase”, parents emphasized the importance of leaving room for hope. The same authors also highlighted that in other phases of communication, parents appreciated when healthcare professionals asked how they preferred to receive information and were able to perceive and value emotional support when it was offered to them [20]. Our findings align with those of other studies exploring the communication needs of parents and professionals, emphasizing the need to bridge the gap between parental expectations and how information is communicated. Healthcare professionals must understand parents’ needs and preferences when delivering difficult news, such as the diagnosis of a rare genetic disease or a complex clinical condition. Parents may feel unfamiliar with the healthcare team, experience uncertainty due to a lack of definitive answers, worry about their child’s health, and fear expressing their own emotions or vulnerabilities [21,22,23]. A clear and empathetic approach fosters shared decision-making, while unclear or rushed communication can harm the relationship between healthcare professionals and parents. In our study, some parents felt welcomed and supported by healthcare professionals during communication, and their expectations were met. In contrast, other parents expressed a need for humility from professionals and stressed the importance of avoiding definitive judgments, which could feel overwhelming. These findings are consistent with those of [23], who evaluated parents’ experiences regarding the communication of their child’s hearing impairment. Parents generally felt satisfied with how the information was conveyed, highlighting the importance of empathy in communication. Most parents perceived the diagnosis as clear and well-explained, while some requested that both parents be present during communication, and others suggested including a family member [23]. In our study, parents also reported significant fatigue, especially after discharge, with many mothers shouldering the responsibility of home care and family organization. This often leads to both physical and emotional exhaustion, a concern noted in other studies on caregivers of children with chronic illnesses [24,25]. Additionally, parents of CMCs expressed concerns about the future and the social integration of their child. Social integration for CMCs is particularly challenging, as parents fear exclusion, bullying, or isolation due to their child’s health condition. Social inclusion is critical for promoting socio-emotional development and reducing discrimination, which benefits both children and parents. Social inclusion involves integrating all children within the same social context, supporting the socio-emotional development of each child, promoting mental health, enhancing respect for diversity, and reducing discrimination [26]. Finally, all CMC participants in the study were treated in an NICU that practices Family-Centered Care (FCC), which included skin-to-skin and kangaroo care. These practices, along with affective touch, have been shown to reduce parental stress and strengthen the parent–child bond [27].

As for the secondary aim of this study, a multidisciplinary NICU team dedicated to the comprehensive care of CMCs is currently being developed. This team will ensure that CMCs receive the highest standard of care throughout their hospital stay, addressing not only their medical needs but also their developmental, emotional, and social well-being. By fostering a collaborative approach, the joint efforts of physicians, nurses, physiotherapist, speech and language therapists, and other support staff are vital in delivering the best possible care for these children, both during their NICU stay and transition home.

Our study has some limitations. The small number of parents interviewed is a result of the difficulty in recruiting CMC participants, as each child has a unique diagnosis, varying medical complexities, and distinct caregiving burdens. Another potential limitation is the absence of a triangulation method in the data analysis, which could have strengthened the reliability and validity of our findings. We acknowledge that these limitations may affect the generalizability of our results. However, this study is one of the few that qualitatively evaluates the impact and experiences of parents of CMCs.

## 5. Conclusions

In conclusion, our study underscores the practical challenges that parents of CMCs face daily, particularly around caregiving, family management, and emotional well-being. A clear, yet hopeful approach to communication is essential for these families. Addressing caregiver fatigue, supporting social integration, and incorporating Family-Centered Care practices, such as affective touch, can significantly reduce stress and strengthen the parent–child bond.

The practical implications of this study suggest that healthcare providers should tailor communication to meet the specific needs of each family, balancing honesty with hope. This personalized approach can help mitigate the emotional burden of caregiving and enhance the parent–child relationship. Furthermore, fostering an empathetic and transparent communication style is key to improving parents’ understanding of complex diagnoses and treatment options, thereby improving their overall experience during a period of intense stress and uncertainty. Ultimately, adopting these practices can lead to more effective family support, better management of CMCs’ care, and a more positive healthcare experience for both parents and children.

## Figures and Tables

**Table 1:** Clinical characteristics of the CMCs.

	Diagnosis
1	Congenital Myotonic Dystrophy SM1
2	Congenital myasthenia
3	Trisomy 18
4	2p21 Microdeletion Syndrome
5	SMA1
6	18q- Syndrome
7	Developmental Epileptic Encephalopathy due to *SPTAN1* gene variant
8	VACTERL Syndrome
9	TRAF7 Syndrome
10	PIK3CA-Related Overgrowth Spectrum (PROS)

SMA: Spinal muscular atrophy.

**Table 2:** Interview guide.

Prenatal period 1.1 During the pregnancy, how were you informed that there was a problem with your baby?
2. Birth 2.1 How did you feel when your baby was admitted to the NICU?
3. Admission 3.1 How was the communication with the professionals who took care of your baby? 3.2 How did you feel when the diagnosis was communicated to you? 3.3 How did you perceive the information that your baby would need medical devices/equipment?
4. Discharge 4.1 How did you feel when you were informed that your baby was ready to be discharged? 4.2. How did you perceive the support network arranged after discharge?
5. At home 5.1 What concerns you most about caring for a child with high-intensity needs at home? 5.2 What aspects, in your opinion, should be implemented regarding the care of children with special needs? 5.3 What is your vision for the future for your child and your family? What do you expect?

**Table 3:** Domains, themes, and excerpts from parents’ experience.

Domains	Themes	Excerpts
1. Parents’ emotions		
	Anxiety and Fear for Survival	M1. I was feeling unwell, overwhelmed by fear and anxiety, and we didn’t know if she would be able to breathe. Even though we couldn’t always be by her side, we quickly understood that A. was in a safe, protected environment at the hospital, which brought us some comfort. F4. I thought that if she couldn’t eat on her own, a gastrostomy would be the best option to improve her quality of life. Mum was terrified, worried that she was gravely ill and might die. I, on the other hand, wasn’t afraid. I felt a sense of calm knowing she was in the hospital, receiving the necessary care. M6. What terrified me most was being asked to sign papers and agree to tests I didnot fully understand—and I could barely remember their names. At that moment, the fear was overwhelming. My mother kept asking, “What’s wrong with her? What are they doing to her?” but I couldn’t offer much of an explanation. It was also a difficult time because of COVID—only one of us could visit at a time, either me or her dad. We didn’t even talk much. It felt like we were both avoiding the situation, like ostriches with their heads in the sand. Day after day, it was as though I was caught in a whirlwind, absorbing some things, understanding others, but losing grip on many details. I haven’t forgotten much, but I remember that, until she came home, her dad and I barely spoke. We were both just … scared.
	Hope and Trust	M1. I found reassuring that just from the admission you told us the true situation, maybe a little bit worse than it actually was. This approach helps to appreciate the improvement and gives you some hope when improvements occur. M6. When delivering news and explaining situations, it’s important to always leave two possible paths: one where things may resolve positively, and one where there might be challenges, and we’ll face them as they come. Never say “obviously”. Bad news must be shared, but it’s equally important to offer hope—to mention that there are people who can help, that new treatments are available, and that while nothing is perfect, we must try to find the positive even in difficult situations. The message should always be the same: stay as positive as possible, and don’t give up right away. Wait and see, because not everyone reacts the same way, and not everyone handles things the same way.
	Guilt and Fatigue	M2. In the beginning, it was very difficult, especially for me. Being with her all the time, I’d wake up in the morning and the first thing I’d see was her, surrounded by all those medical devices. At first, she had to be fed around the clock, and I had to constantly switch between different formulas. I felt a bit down because it was so different from the routine of going to the hospital every day, where I had some free moments—like taking a walk, for example. But in the early days at home with her, I couldn’t leave the house, and even inside, I had to stay in the same room with her because she couldn’t be moved to the other rooms. It was hard for me. Yes, we had support, but I still struggled. I felt like I was carrying the weight of everything, because her dad was working and only came home in the evening. I didn’t handle it well, but I found a way to gather my strength. M8. [I felt] anxious, I believe that everything is in God’s hands, but I was afraid I wouldn’t be able to help her.
2. Relationship with healthcare professionals		
	Expectations	M4. You couldn’t have done more than what you did. Even in terms of information, you were clear. Although there is the language barrier, when it was necessary, you explained things clearly, even 2–3 times. M6. It’s all about the initial approach with parents, who often don’t even know why they ended up here. It’s important to clearly explain what might be wrong with their child, why certain tests are being conducted, and what they could potentially reveal. Personally, seeing my psychotherapist really helped me. There was a service available here, but I never used it since I had my own therapist, so I continued seeing her. However, I believe psychological support for parents is essential. It’s great for those who already have a therapist, but for those who don’t, accessing the psychological services offered by the unit would definitely be a comfort. Fears emerge day by day, and they tend to grow and amplify... In the beginning, everything seemed so tragic.
	Frustrations	F3. “… but in […], we felt more supported. In […], they asked us, ’What do you expect from us?’ And we said: to do everything possible, day by day. AP has always managed to overcome obstacles by ’climbing the ladder’ on her own; she just needs support. Here, I sensed a certain pride in not referring to other centers for comparisons. In the end, we reached an impasse, and I signed. What’s disappointing is the lack of openness. Instead, there should be collaboration. We know it’s a tough diagnosis, but we want the best; there has to be cooperation with the parents. [Father 3]
3. Relationship within the couple		
	Different perception of mother and father	M9. He is angry because, by nature, he is someone who always wants to solve everyone’s problems. […] He is very authoritative, and not being able to help the most important thing in his life makes him think about it every day, wondering whether he should try this or that. It’s fine to stay determined, but you can’t always expect answers, especially since new challenges with M. come up every now and then. I had this conversation with him when M was one year old, but he still hasn’t accepted it. Meanwhile, I spend the whole day with M, while he only sees her for 2–3 h in the evening. [Mother 9]
4. Outlook of the future		
	Daily life at home	F3. At home, we managed on our own. They even gave us incorrect instructions, and because of that, we waited too long to take her to the ER, and she went into cardiac arrest. It’s important to really know the child. Now, we have a record where we note down everything we do, and this helps us understand what’s happening. We’ve been doing it for 8 years, and it’s essential. We do parent-led education; she doesn’t go to school, but we’re in contact with the school, and every year we provide a report. So, AP ’exists’ for the school too. But going to school is a hassle—with the gastrostomy, the CPAP, and then she sleeps in the morning. Her interaction is like that of a newborn. … Every family has its own path, its own journey; this is ours.
	Social integration	M6. As long as she’s small, she stays in the infant car seat, which makes it easier—she seems like all the other kids, no one asks questions, no one stares. It’s as she grows that the difference becomes more noticeable. For example, my husband didn’t want me to take her out with the hearing aids. He didn’t want others to see them; he was very self-conscious about everything. Before, it was a world we definitely didn’t know and didn’t even know existed, but when you have a child with special needs, a whole new world opens up. And since I was always the one going out, that world opened up to me. I used to tell him that it would do him good to come and see her, but he always struggled with it, much more than I did. F7. Also, because when we went to nursery, we had to compare her with the other children, and that hurts. We love her infinitely, but it hurts to see this beautiful little girl who has millions and millions of problems.

M: mother, F: father.

## Data Availability

The data presented in this study are available on request from the corresponding author due to privacy reasons.
